# Dual Regulatory Roles of Human AP-Endonuclease (APE1/Ref-1) in CDKN1A/p21 Expression

**DOI:** 10.1371/journal.pone.0068467

**Published:** 2013-07-16

**Authors:** Shiladitya Sengupta, Sankar Mitra, Kishor K. Bhakat

**Affiliations:** 1 Department of Biochemistry and Molecular Biology, University of Texas Medical Branch, Galveston, Texas, United States of America; 2 Sealy Center for Molecular Medicine, University of Texas Medical Branch, Galveston, Texas, United States of America; University of Texas Health Science Center at San Antonio, United States of America

## Abstract

The human AP-endonuclease (APE1/Ref-1), an essential multifunctional protein involved in repair of oxidative DNA damage as well as in transcriptional regulation, is often overexpressed in tumor cells. APE1 was earlier shown to stimulate p53’s DNA binding and its transactivation function in the expression of cyclin-dependent kinase inhibitor p21 (CDKN1A) gene. Here, we show APE1’s stable binding to p53 *cis* elements which are required for p53-mediated activation of p21 in p53-expressing wild type HCT116 cells. However, surprisingly, we observed APE1-dependent repression of p21 in isogenic p53-null HCT116 cells. Ectopic expression of p53 in the p53-null cells abrogated this repression suggesting that APE1’s negative regulatory role in p21 expression is dependent on the p53 status. We then identified APE1’s another binding site in p21’s proximal promoter region containing *cis* elements for AP4, a repressor of p21. Interestingly, APE1 and AP4 showed mutual dependence for p21 repression. Moreover, ectopic p53 in p53-null cells inhibited AP4’s association with APE1, their binding to the promoter and p21 repression. These results together establish APE1’s role as a co-activator or co-repressor of p21 gene, dependent on p53 status. It is thus likely that APE1 overexpression and inactivation of p53, often observed in tumor cells, promote tumor cell proliferation by constitutively downregulating p21 expression.

## Introduction

The mammalian apurinic/apyrimidinic-endonuclease (APE1/Ref-1), a ubiquitous and multifunctional protein, was initially characterized as a central player in DNA base excision repair (BER) pathway [1], [2], [3]. APE1 repairs apurinic/apyrimidinic (AP) sites and DNA single-strand breaks induced by reactive oxygen species and alkylating agents, and their oxidation products generated in the genome either spontaneously or after excision of oxidized and alkylated bases by DNA glycosylases. APE1 may also function in the nucleotide incision repair pathway by incising DNA 5′ to oxidatively damaged bases including 5,6-dihydrothymidine and alpha-2′deoxyadenosine [4], [5].

Apart from its key role in DNA BER, mammalian APE1 possesses two unique and apparently distinct transcriptional regulatory functions. It acts as a reductive activator of several transcription factors including c-Jun, p53, NF-κB p50, HIF1-α etc and was independently named redox effector factor-1 (Ref-1) [6], [7], [8], [9]. APE1 could also act as a redox-independent *trans*-acting factor during Ca^2+^-dependent downregulation of parathyroid hormone (PTH) gene [10]. APE1 was identified in the *trans*-acting complex that binds to negative Ca^2+^-response elements (nCaRE-A and B) in PTH promoter [10]. Subsequently, the presence of nCaRE-B element and binding of APE1 to this element was also shown in the human renin gene promoter [11]. Recently, we showed that APE1 also acts as an nCaRE-independent negative regulator for mouse renin gene expression via recruitment of HDAC1 co-repressor complex to renin promoter [12]. APE1 is acetylated *in-vitro* and in cultured cells at Lys 6/7 by p300 [13] and deacetylated by SIRT1 [14]. We showed that acetylation of APE1 stimulates formation of the nCaRE-B complex in PTH promoter which contains hnRNP-L and HDAC1 [13]. Furthermore, others in collaboration with us also reported the involvement of APE1 in the transcriptional regulation of Bax [15], Egr-1-mediated PTEN [16] and IL-6-inducible STAT3-mediated acute-phase reactant gene expression [17]. APE1 was found to be essential in mouse and also in cultured human and mouse cells [18], [19], [20]. We also showed that both acetyl acceptor Lys 6/7 and the active His 309 residue in APE1 are essential for cell survival [20]. APE1 was found to be ubiquitinated by MDM2 at specific N-terminal Lys residues [21] and phosphorylated by CDK5 at Thr 233 [22] which enhanced its ubiquitination and also modulates its gene regulatory functions [23].

APE1 is often overexpressed in tumor tissues and cancer cells of diverse origin including ovarian, cervical, prostate, glioma, head and neck, germ-cell, non-small-cell lung carcinoma etc and its overexpression is associated with tumor cells’ resistance to various anticancer drugs [8], [24], [25], [26]. Targeted knockdown of APE1 in mammalian cells or its functional impairment enhances apoptosis, inhibits cell proliferation and sensitizes cells to a variety of genotoxic agents eg. MMS, H_2_O_2_, bleomycin, TMZ, BCNU, etoposide, cisplatin and doxorubicin etc. [6], [25], [27], [28], [29], [30], [31], [32], [33]. We made a novel observation on APE1’s transcriptional regulatory function and associated drug resistance. We showed that APE1 and its acetylated form activate multiple drug resistance gene MDR1 by interacting with p300/YB-1 and RNA pol II and promoting their recruitment to the MDR1 promoter [34].

The tumor suppressor p53 is a transcriptional activator that plays an essential role in DNA damage response by inducing cell cycle arrest, senescence and/or apoptosis [35], [36]. p53 triggers cell cycle arrest at G1 phase by transactivating cyclin-dependent kinase inhibitor 1A (CDKN1A)/p21 gene which causes cell cycle arrest and suppresses cell proliferation [37], [38], [39]. Earlier studies showed that APE1 stimulates DNA binding activity of p53 *in-vitro* by both redox-dependent and independent mechanisms [40] and its transactivation function in p21 regulation [41]. On the other hand, global gene expression profile analyses by Tell group in collaboration with us identified p21 as one of the genes that is upregulated in APE1-knowkdown HeLa cells in which p53 is nonfunctional [42]. Subsequently, Jiang *et al* also documented upregulation of p21 expression and impairment of cell cycle progression and proliferation by silencing APE1 in pancreatic cell lines [43]. We addressed this paradox by analyzing the role of APE1 in p21 expression in p53-expressing colon carcinoma HCT116 cells (HCT116^WT^) and its isogenic p53-null cells (HCT116^p53null^). We report here that APE1 functions as a constitutive co-repressor for p21 gene by its association with transcription factor AP4. However, APE1’s co-repressor function is overridden in the presence of p53 which after binding to APE1 activates the p21 gene. We have thus provided the first evidence for dual and opposite transcriptional co-regulatory roles of APE1 in controlling p21 expression which is dependent on p53 status.

## Materials and Methods

### Cell lines, Plasmids, siRNA and Transfection

Human colorectal adenocarcinoma HCT116^WT^ and HCT116^p53null^ lines [39] were kind gifts from Dr B. Vogelstein, Johns Hopkins University School of Medicine and were cultured in McCoy’s 5A medium (Gibco-BRL) supplemented with 10% fetal calf serum (Sigma), 100 U/ml penicillin and 100 µg/ml streptomycin (Gibco-BRL). Exponentially growing cells were treated with 10 µM etoposide and harvested after 5 hours. Wild Type (WT) APE1/N-terminal 42 amino acid deleted (NΔ42) mutant APE1 expression plasmid in PCDNA3 backbone, PCMV 5.1 FLAG-tagged expression plasmid for WT APE1/N-terminal 33 amino acid deleted (NΔ33) mutant APE1 were described elsewhere [12], [27], [34]. p53 expression plasmid used in this study was kindly provided by Dr. A. L. Levine (University of Medicine and Dentistry, New Jersey, New Brunswick). siRNAs specific for APE1, AP4 mRNA and universal control siRNA were obtained from Sigma. Exponentially growing cells were transfected with Lipofectamine 2000 (Invitrogen) following manufacturer’s protocol and harvested for RNA isolation and RT-PCR, luciferase activity assay, chromatin immunoprecipitation (ChIP) assay, co-immunoprecipitation (co-IP) assay and Western analysis as required.

### Co-Immunoprecipitation (Co-IP) and Western Analysis

Immunoprecipitation (IP) was done with mouse monoclonal α-FLAG M2 antibody-conjugated agarose beads (Sigma; # A2220) in extracts of cells transfected with FLAG tagged constructs as described previously [13], [27], [44], [45]. IP was also done with mouse monoclonal α-APE1 antibody (Novus Biologicals; # NB100–116) or control IgG and protein A/G Plus agarose beads (Santa Cruz; # sc-2003) in the pre-cleared extracts of control and experimental cells. The immunoprecipitated proteins were resolved in SDS-PAGE and identified by Western analysis with the indicated antibodies; HRP-conjugated mouse α-FLAG (Sigma; # 8592), mouse monoclonal α-p53 (Millipore, # CS200578) and goat α-AP4 (Santa Cruz; # sc-18593). Depletion or overexpression of APE1 in experimental and control cells were detected by Western analysis of 48–96 hours-post transfected cells with α-APE1 antibody [46].

### Luciferase Assay

Cells were co-transfected with p21-promoter luciferase reporter construct (kindly provided by Dr. A. L. Levine) and expression plasmid for APE1 or empty vector and luciferase activity in the extracts of 36–48 hours-post transfected cells was measured in a luminometer (AutoLumant LB 953; Berthold) using the luciferase assay kit (Promega). The luciferase activity was normalized with respect to total protein content of the lysates.

### RNA Isolation and Real Time RT-PCR Assay

Total RNA was isolated from cells with Qiagen RNeasy mini kit followed by DNase 1 (NEB) treatment and proceeded for cDNA synthesis using Superscript III first-strand synthesis kit (Invitrogen). AP4 and p21 expression in the samples were analyzed by SYBR GREEN-based Real Time PCR (7000 Real-Time PCR System; Applied Biosystems) using SYBR Premix Ex Taq (TaKaRa) and primers (Table 1) appropriate for p21, AP4 or HPRT1 expression (internal control). Data were represented as relative quantitation with respect to the reference samples set at 1 based on 2^−ΔΔCT^ method.

**Table 1:** List of primers.

p21 promoter: p53 binding site 1 ChIP PCR	F: caggctgtggctctgattgg
	R: ttcagagtaacaggctaagg
p21 promoter: p53 binding site 2 ChIP PCR	F: ggtctgctactgtgtcctcc
	R: catctgaacagaaatcccac
p21 proximal promoter ChIP PCR	F: ggtgcttctgggagaggtgac
	R: tgacccactctggcaggcaag
p21 RT-PCR	F: gcagaccagcatgacagattt
	R: ggattagggcttcctcttgga
AP4 RT-PCR	RealTimePrimers.com
HPRT1 RT-PCR	RealTimePrimers.com

### Chromatin Immunoprecipitation (ChIP) Assay

ChIP assay was performed after double crosslinking of cells with disuccinimidyl glutarate and formaldehyde [47], with Magna ChIP Protein A Magnetic beads (Millipore, # 16–661) using the following antibodies: α-APE1 (Novus Biologicals), α-AP4 (Santa Cruz), α-p53 (Millipore) or control IgG (Santa Cruz) as described previously [12], [27], [34]. The immunoprecipitated purified DNA was then subjected to SYBR GREEN-based Real Time PCR with primers (Table 1) for p21 distal promoter containing p53 binding sites and proximal promoter region containing AP4 binding sites. For re-ChIP assay, after the first IP was performed with α-APE1 antibody, the second IP was performed in the eluents with α-AP4 or α-p53 antibody. Data were represented as relative enrichment with respect to IgG control based on 2^−ΔCT^ method.

### Statistics

Data are represented as ± STDEV of two or more independent experiments. The significance of differences between different groups was determined by Student’s T-Test and p value less than 0.05 was considered significant (represented as * in the histograms).

## Results

### APE1 Activates p21 Expression in p53-expressing Cells

**Figure 1:**
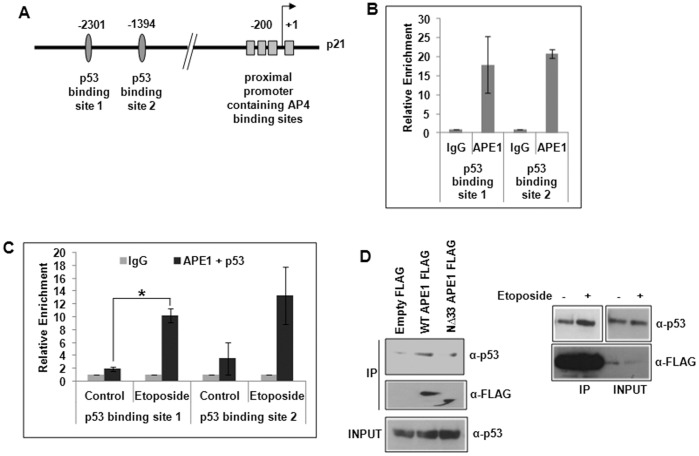
Association of p53 and APE1 on p53-binding sites in p21 promoter. (**A**) p21 promoter structure showing p53 and AP4 binding sites. (**B**) ChIP Real Time PCR analysis showing relative enrichment (2^−ΔCT^) of APE1-immunoprecipitated DNA over that from control IgG in p21 promoter regions containing p53 binding sites 1 & 2 in HCT116^WT^ cells. (**C**) Re-ChIP analysis (first IP with α-APE1 and the second IP with α-p53 antibody) showing simultaneous recruitment of APE1 and p53 in control vs. etoposide treated cells; *: p value <0.05 (n = 2) calculated based on APE1/p53 enriched DNA from control vs. etoposide treated cells. (**D**) Western analysis of FLAG immunoprecipitate (IP) to detect APE1-associated p53 and FLAG (APE1) from empty vector vs. FLAG-tagged WT APE1 or FLAG-tagged NΔ33 APE1 transfected HCT116^WT^ cells (left panel) and from control vs. etoposide-treated WT APE1-FLAG transfected cells (right panel).

**Figure 2:**
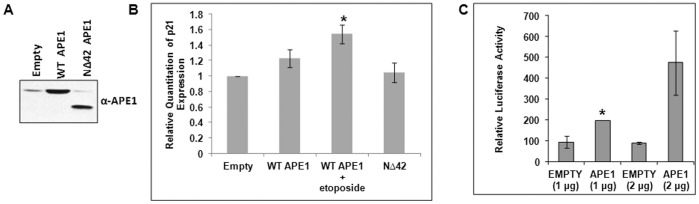
Effect of APE1 on p21 activation in p53 WT cells. (**A**) Western analysis for APE1 level in WT APE1 and NΔ42 APE1 overexpressing cells. (**B**) Real Time RT-PCR analysis showing relative quantitation of p21 transcript level in etoposide-treated WT APE1-overexpressing HCT116^WT^ cells and NΔ42 APE1 overexpressing cells; *: p value <0.05 (n = 2) calculated from control (empty vector transfection) vs. WT APE1 overexpression, WT APE1 overexpression along with etoposide treatment or NΔ42 overexpression. (**C**) Luciferase activity in cells co-transfected with empty or WT APE1-expression vector and p21 promoter-luciferase construct; *: p value <0.05 (n = 2) calculated from control (empty vector transfection) vs. APE1 overexpression.

Earlier studies have shown that APE1 is a potent activator of p53’s DNA binding *in-vitro* via both redox-dependent and independent mechanisms and also regulates its transactivation function in p21 expression [40], [41]. To the best of our knowledge, we showed for the first time using chromatin immunoprecipitation (ChIP) assay APE1’s stable association with the endogenous p21 promoter sequence that includes p53-binding sites (Fig. 1A & B) in HCT116^WT^ cells. Re-ChIP analysis (Fig. 1C) further showed that APE1 and p53 were simultaneously bound to these regions; the binding was enhanced by etoposide treatment, a DNA topoisomerase II inhibitor drug that causes double-strand breaks [48]. Additionally, co-immunoprecipitation (co-IP) assay with anti-FLAG antibody showed a significant presence of p53 in FLAG-tagged wild-type (WT) APE1 immunoprecipitate (IP) and less in FLAG-tagged N-terminal 33 amino acid deleted (NΔ33) mutant APE1 (Fig. 1D; left panel). The level of p53 in FLAG-tagged WT APE1 IP was enhanced by etoposide treatment (Fig. 1D; right panel). Real Time RT-PCR analysis showed that ectopic expression of WT APE1 enhanced p21 expression in presence of etoposide (Fig. 2B). This activation was not observed with NΔ42 APE1 mutant (Fig. 2B) as compared to WT APE1; the expression level of WT and NΔ42 APE1 is shown in Fig. 2A. This indicates that APE1’s N-terminal region is necessary for p21 activation. Using p21 promoter-dependent luciferase reporter assay, we also showed that APE1 activates p21 promoter activity in a dose-dependent manner (Fig. 2C), thus establishing the role of APE1 in p21 activation in WT p53-expressing cells. Taken together, our observations implicate APE1’s co-activator role in p21 expression in WT p53 cells.

### APE1 Negatively Regulates p21 Expression in the Absence of p53

**Figure 3:**
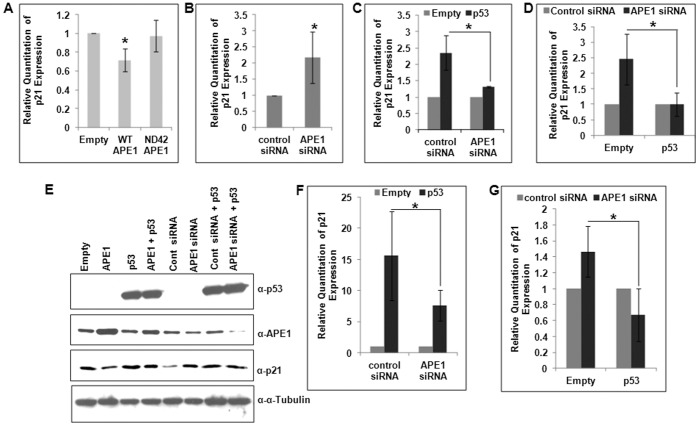
Repression of p21 by APE1 in p53-null cells and effect of ectopic p53 in this repression. (**A & B**) Real Time RT-PCR analysis showing relative quantitation of p21 transcript level in **(A**) HCT116^p53null^ cells with WT and NΔ42 APE1 overexpression; *: p value (n = 4) calculated from control (empty vector transfection) vs. WT or NΔ42 APE1 overexpression, and (**B**) control (control siRNA) vs. APE1-depleted HCT116^p53null^ cells; *: p value <0.05 (n = 4) calculated from control vs. APE1-depleted cells. (**C**) Effect of ectopic p53 expression on p21 transcript level in control vs. APE1-depleted HCT116^p53null^ cells. First, cells were transfected with control siRNA or APE1 siRNA, the next day both the cell types were again transfected with empty vector or p53 expression vector and after 48 hrs the cells were harvested; signal from empty vector transfection in both control and APE1-depleted cells were set as reference samples; *: p value <0.05 (n = 3) calculated based on the effect of ectopic p53 expression over empty vector transfection in control vs. APE1-depleted cells. (**D**) Effect of APE1 depletion in control (empty vector transfected) vs. ectopic p53-expressing HCT116^p53null^ cells; the same experiment was performed as in C but analyzed differently; signal from control siRNA-transfected cells in both empty vector transfected and ectopic p53 expressing cases were set as reference samples; *: p value <0.05 (n = 3) calculated based on the effect of APE1-depletion in empty vector transfected vs. ectopic p53 expressing cells. (**E**) Representative Western analysis of p53, APE1, p21 and α-Tubulin levels in the same HCT116^p53null^ cells as in B–D. (**F & G**) Real Time RT-PCR analysis of p21 level in Saos2 cells as in C & D. *: p value <0.05 (n = 2).

In contrast to our results about APE1’s involvement as a co-activator in p53-mediated p21 activation in HCT116^WT^ cells, we observed the opposite role of APE1 in the isogenic p53-null (HCT116^p53null^) cells. Overexpression of WT APE1 but not NΔ42 mutant decreased endogenous p21 mRNA level (Fig. 3A). Furthermore, APE1 downregulation enhanced p21 expression (Fig. 3B). These results suggest that APE1 can regulate p21 expression both positively and negatively, depending on the status of p53 in the cell. We then tested the effect of ectopic p53 in APE1’s regulation of p21 expression in p53 null cells. As expected ectopic expression of p53 increased p21 level in these cells, and interestingly we could not observe any significant effect of p53 expression on p21 activation in APE1-downregulated cells (Fig. 3C). The reference samples in both control cells and APE1-depleted cells are empty vector transfected cells and the effect of p53 overexpression in these two cell types were measured. Because APE1 had opposite effects on p21 expression in HCT116^WT^ vs. HCT116^p53null^ cells, we asked whether ectopic expression of p53 in HCT116^p53null^ cells could abrogate APE1’s repressor function. In empty vector transfected cells APE1-depletion could activate p21 mRNA level and this activation was inhibited in ectopically p53-expressing cells (Fig. 3D). In both empty vector transfected and ectopically p53-expressing cells, the reference samples were with control siRNA transfection and the effect of APE1 depletion was measured. The effect of APE1 overexpression and depletion in p53-null and ectopically-expressing cells on p21 regulation was also reflected to some extent at the protein level (Fig. 3E). To confirm that APE1’s repressor activity for p21 expression in the absence of p53 is a general phenomenon, we used another p53-null cell of different origin, namely Saos-2 osteosarcoma line. Fig. 3F showed that ectopic expression of p53 activated p21 expression which was inhibited by APE1 depletion (as observed for HCT116^p53null^ cells in Fig. 3C). On the other hand, Fig. 3G showed that APE1 downregulation in these p53-negative cells enhanced p21 mRNA level and this activation was prevented by ectopic p53 (as observed for HCT116^p53null^ cells in Fig. 3D). Thus, the negative regulatory role of APE1 for p21 expression is evident only in the absence of p53. These results together indicate that APE1 can function both as a co-activator or a co-repressor for p21 expression which is dependent on p53 status.

### APE1 Associates with AP4 on the p21 Promoter

**Figure 4:**
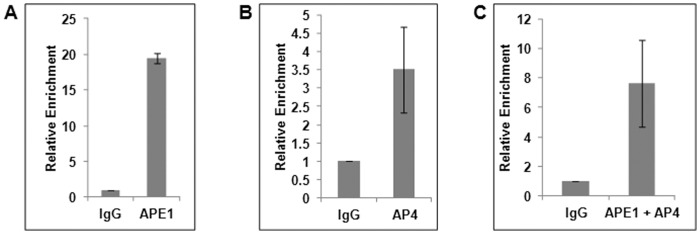
Association of APE1 and AP4 in p21 proximal promoter region. (**A & B**) ChIP Real Time PCR analysis showing relative enrichment of (**A**) APE1 and (**B**) AP4 in p21 proximal promoter containing AP4-responsive E-Box elements in HCT116^p53null^ cells. (**C**) Re-ChIP (first IP with α-APE1 and the second IP with α-AP4 antibody) analysis showing simultaneous recruitment of APE1 and AP4 in this promoter region.

In order to elucidate the mechanism of APE1’s co-repressor function in p21 expression, we analyzed our genome-wide ChIP-on-Chip/ChIP-sequencing data (Sengupta *et al*, unpublished) which showed APE1’s binding to both p21 proximal and distal (containing p53 binding sites) promoter sequences (Fig. 1A). Close inspection of the p21 gene regulatory regions identified potential response elements for a wide range of *trans*-activators and *trans*-repressors [37], [49]. AP4 was identified earlier to be a potent repressor in p21 regulation where it binds to E-box elements located in the proximal promoter [50], [51]. Interestingly, using unbiased proteomics approaches, Ku *et al.* identified APE1 to be one of the potential AP4-interacting proteins bound to the E-box sequence in the HDM2 promoter [52]. We then tested the possibility that APE1 could act as AP4’s co-repressor for p21 via stable association on the p21 promoter. ChIP analysis showed constitutive binding of both APE1 and AP4 to the p21 proximal promoter region containing AP4 binding sites (Fig. 4A & B). Furthermore, simultaneous binding of both AP4 and APE1 to this region was shown by re-ChIP analysis (Fig. 4C). These results strongly suggest that APE1’s negative regulatory role in p21 expression could possibly be due to its association with the repressor AP4.

### APE1 is Essential for AP4-mediated p21 Repression

**Figure 5:**
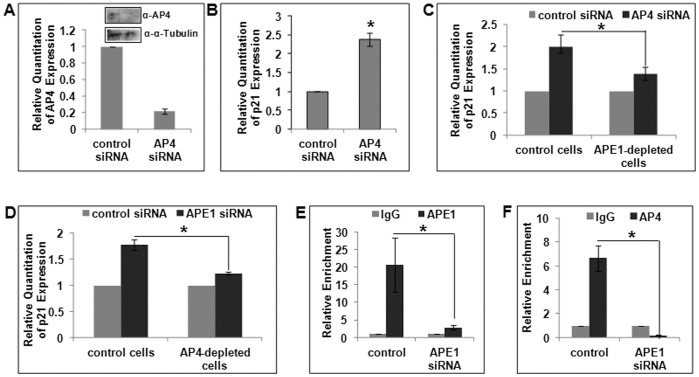
Mutual dependence of APE1 and AP4 for p21 repression. (**A & B**) Real Time RT-PCR analysis in HCT116^p53null^ cells showing relative quantitation of (**A**) AP4 transcript level after AP4-depletion by siRNA transfection (72 hrs); Western analysis of AP4 level shown in the inset, (**B**) p21 transcript level in the same cells as in A; *: p value <0.05 (n = 3) calculated from control vs. AP4-depleted cells. (**C**) Effect of AP4-depletion in control vs. APE1-depleted HCT116^p53null^ cells. Cells were first transfected with control siRNA or APE1 siRNA, the next day both cell types were re-transfected with control or AP4 siRNA and after 72 hours the cells were harvested; *: p value <0.05 (n = 2) calculated based on the effect of AP4 knockdown in control vs. APE1-depleted cells. (**D**) Effect of APE1-depletion in control vs. AP4-depleted HCT116^p53null^ cells; same experiment was performed as in C but analyzed differently; *: p value <0.05 (n = 2) calculated based on the effect of APE1 knockdown in control vs. AP4-depleted cells. (**E & F**) ChIP Real Time PCR analysis showing relative enrichment of (**E**) APE1 and (**F**) AP4 in p21 proximal promoter in control vs. APE1-depleted HCT116^p53null^ cells; cells were transfected with control or APE1 siRNA and ChIP assay was performed after 72–96 hours; *: p value <0.05 (n = 4) calculated based on relative enrichment of APE1 or AP4 in control vs. APE1-depleted cells.

We then explored APE1’s regulatory role in p21 repression. First, we confirmed AP4’s repressor role in p21 expression (Fig. 5B) in p53 null cells after siRNA-mediated depletion of AP4 (Fig. 5A, Western analysis for AP4 level in these cells shown in the inset). Next, we analyzed the effect of AP4 depletion on p21 activation in control and APE1-downregulated cells. Depletion of endogenous AP4 activated p21 expression in control cells, and interestingly this AP4-knockdown mediated p21 activation was inhibited in APE1-depleted cells (Fig. 5C). The reference samples in both control and APE1-depleted cells are control siRNA transfected cells and the effect of AP4 depletion was measured. This suggests that APE1 is required for AP4-mediated repression of p21. Next, the same set of samples was analyzed differently to examine the effect of APE1 depletion in control and AP4 depleted cells. Again, as expected APE1 depletion activated p21 expression in control cells, and in AP4-depleted cells, APE1 depletion could not activate p21 expression to the same efficiency (Fig. 5D). The reference samples in both control and AP4-depleted cells are control siRNA transfected cells and the effect of APE1 depletion was measured. These results imply that APE1 and AP4 are mutually dependent for p21 repression.

We then tested whether APE1 knockdown inhibits AP4’s recruitment to p21 proximal promoter. APE1-depleted cells exhibited reduced recruitment of both APE1 (Fig. 5E) and AP4 (Fig. 5F) as compared to control cells. Thus, taken together these data suggest the critical role of APE1 in AP4-mediated repression of p21.

### p53 Interferes with APE1/AP4 Association and Promoter Recruitment

**Figure 6:**
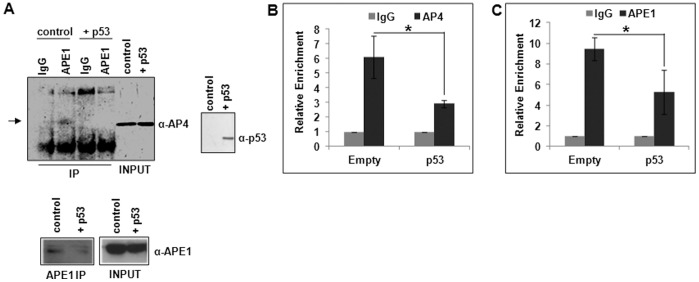
Effect of p53 in APE1/AP4 association and their recruitment to p21 promoter. (**A**) Western analysis of APE1-IP to detect APE1-associated AP4 in control vs. ectopic p53 expressing HCT116^p53null^ cells; AP4, APE1 and p53 levels also shown in input samples. (**B & C**) ChIP Real Time PCR analysis showing relative enrichment of (**B**) AP4 and (**C**) APE1 in p21 proximal promoter in control vs. ectopic p53-expressing HCT116^p53null^ cells; cells were transfected with empty vector or p53-expression vector and ChIP assay was performed after 48 hours; *: p value <0.05 (n = 5) calculated based on the relative enrichment of AP4 or APE1 in control vs. ectopic p53 expressing cells.

Our observations indicating APE1’s dual and contradictory roles in p53-mediated activation and AP4-mediated repression of p21 gene have significant clinical implications in that a predominant fraction of tumor cells have inactive p53. Hence it is important to determine if the effect of WT p53 dominates during DNA damage-induced p21 activation by preventing APE1-dependent AP4 repressor activity. We thus tested the hypothesis that p53 interferes with AP4/APE1-association as follows. In co-IP experiment, we observed reduced level of AP4 in the APE1 IP from HCT116^p53null^ cells with ectopic p53 expression, compared to control cells (Fig. 6A). ChIP assay also showed that recruitment of AP4 and APE1 to p21 proximal promoter was inhibited in these cells (Fig. 6B & C). These data together suggest that p53 abrogates APE1/AP4 association, their promoter recruitment and thereby inhibiting their repressor function.

## Discussion

Stress-induced p53-mediated activation of p21 causes cell cycle arrest at specific stages (G1/S or G2/M) of the cell cycle by inhibiting cyclin-dependent kinases and also interferes with PCNA-dependent DNA polymerase activity thereby inhibiting DNA replication [37], [39], [53], [54], [55]. Deregulation of p21 expression has been linked with turmorigenesis and resistance of tumor cells to anti-cancer drugs [37], [56], [57]. Thus, elucidating the underlying mechanism of p21 gene regulation related to cell proliferation and tumorigenesis is important. In this study, we have unraveled the dual role of APE1 as a co-activator or a co-repressor in p21 regulation which is dependent on the p53 status. We have provided evidences of APE1’s stable association with p53 on p53 binding sites in p21 promoter which is required for its co-activator function in p53-mediated transactivation of p21 gene. Most importantly, we made the novel observation that APE1 also serves a co-repressor of p21 gene in the absence of p53. We further explored that APE1-mediated repression of p21 is driven by AP4, a repressor of p21 that binds to E-box elements in the proximal p21 promoter, distinct from the p53 binding sites. Thus, we have unraveled a likely mechanism of how APE1’s overexpression often observed in tumor cells, is linked to sustained cell proliferation via constitutively downregulating p21 expression. Our studies have also provided the first evidence for dual and opposite transcriptional co-regulatory roles of APE1 in controlling p21 expression which is dependent on status of p53.

p53 directly binds to two highly conserved p53 binding sites present in p21 promoter [38], [58]. While stimulation of p53’s *in-vitro* DNA binding by APE1 was shown earlier, [40], we have provided first ChIP-based direct evidence that APE1 and p53 remain simultaneously bound as a stable complex to the p21 promoter *in-cell* which was further enhanced after genotoxic stress. Our Co-IP analysis showed the presence of p53 in APE1 immunocomplex which was also enhanced by genotoxic stress. Interestingly, APE1’s activating role in p21 regulation requires its N-terminal region. This positively charged, intrinsically disordered region of mammalian APE1 absent in the bacterial prototype X^th^ [7], [59] is necessary for its interactions with various binding partners e.g. XRCC1 [60], YB1 [27], NPM1 [61], STAT3 [17] etc. It is likely that APE1 has multiple functions with respect to p53 binding: stable binding to p53 making the protein accessible for redox regulation and also facilitating its loading to the promoter and thus functions as a direct *trans* co-activator.

The key observation in this study is that, in stark contrast to APE1’s co-activator role for p21 expression in p53-expressing cells, APE1 overexpression represses p21 expression in p53-null cells and knockdown of endogenous APE1 activates p21 expression. To elucidate this repressor activity, we found that APE1 is constitutively associated with p21 proximal promoter regardless of p53 status of the cells. Based on a series of experiments, we concluded that APE1 plays a pivotal role in p21 downregulation by acting as a co-repressor that facilitates the binding of AP4 repressor complex to p21 proximal promoter. Our co-IP assay (Fig. 6A) and ChIP/re-ChIP analysis (Fig. 4A–C) provided strong evidence for simultaneous stable association of APE1 with AP4 on p21 promoter and that both are dependent on each other for p21 repression (Fig. 5C–F). However, APE1’s co-repressor function is apparent only in the absence of p53 because ectopic expression of p53 in p53-null cells abrogates APE1-mediated p21-repression (Fig. 3D, E & G). These cells also exhibited reduced amount of AP4 associated with APE1 (Fig. 6A), which is also reflected at reduced promoter occupancy of AP4 and APE1 (Fig. 6B & C) and consequent repression function (Fig. 3D, E & G). Thus, it is likely that the presence of p53 reduces AP4 *cis*-element binding presumably by squelching the available APE1 necessary for loading AP4 repressor complex to the promoter. Thus, APE1’s co-activator or co-repressor function for p21 expression is dependent on p53 status of the cells. These novel findings on APE1’s co-repressor function support a previous study which also showed that APE1 depletion increased p21 expression in p53-inactive HeLa cells [42]. Jiang *et. al.* also documented upregulation of p21 expression and impairment of cell cycle progression by silencing APE1 expression in pancreatic cells PANC-1 and PaCa2 [43], both of which express non-functional mutant p53 protein [62]. Thus, the dual regulatory roles of APE1 as dissected in this study may explain the basis of why APE1 downregulation had opposite effects on p21 expression in different cell lines.

**Figure 7:**
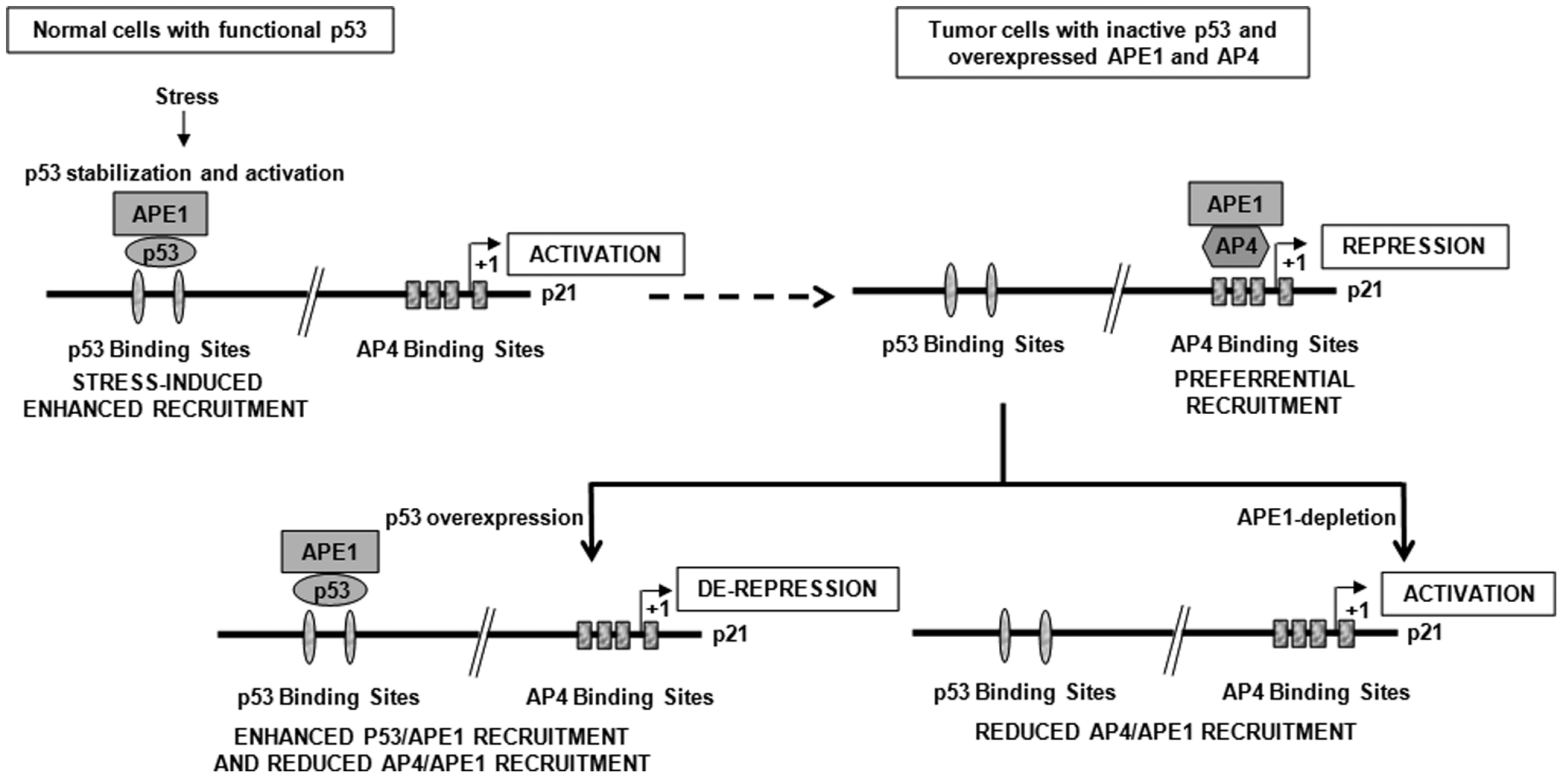
A model for crosstalk between APE1 with p53 and AP4 in p21 gene regulation.

Overexpression of APE1 is a commonly observed phenomenon in tumor tissues and cancer cell lines with associated drug resistance which could be due to its both repair and regulatory functions [6], [8], [25], [28], [29], [30], [31], [33], [43], [63]. In an independent study we showed negative regulation of APE1 by WT p53 [64] which could contribute to APE1’s overexpression in p53-inactive tumors. Tumor cells have consistent overexpression of both APE1 and AP4 [50], [65], [66], [67] with repressed p21 [37], [56], [57]. We explored the complex regulation of p21 gene expression connecting APE1 and AP4 with p53. This study indicating APE1’s dual and contradictory roles in p53-mediated activation and AP4-mediated repression of p21 gene have significant clinical implications in that a predominant fraction of tumor cells have inactive p53. We propose a model for the transcriptional regulation of p21 by APE1 (Fig. 7). In non-tumorigenic replicating cells which maintain a low level of p53 and p21, genotoxic challenge activates and stabilizes p53 and upregulates p21 expression with the help of APE1 as a co-activator and arrests cell proliferation. In p53-inactive tumor cells where both APE1 and AP4 levels are high, APE1’s constitutive co-repressor function maintains repressed p21 level for sustained cell proliferation. Overexpression of p53 or APE1-knockdown in these p53-inactive tumors could activate p21 gene and arrest tumor growth. Thus it is likely that the presence of WT p53 dominates during DNA damage-induced p21 activation by preventing APE1-dependent AP4 repressor activity. Further studies are necessary to establish a direct correlation of APE1 levels with p21 expression in tumor tissues having different p53 status: wild type, null, inactive or oncogenic mutations. Targeting APE1’s transcriptional co-regulatory function or its redox functions by small-molecule inhibitors is an emerging concept that is receiving much deserved attention for sensitizing cancer cells to DNA damaging agents [68], [69]. Thus screening tumor patients for p53 status would enable proper management of APE1-targeted cancer therapy through pharmacological modulation of its transcriptional regulatory activities.
